# Subconcussive impacts and imaging findings over a season of contact sports

**DOI:** 10.2217/cnc-2016-0003

**Published:** 2016-08-16

**Authors:** Elizabeth M Davenport, Jillian E Urban, Fatemeh Mokhtari, Ervin L Lowther, John D Van Horn, Christopher G Vaughan, Gerard A Gioia, Christopher T Whitlow, Joel D Stitzel, Joseph A Maldjian

**Affiliations:** 1 Advanced Neuroscience Imaging Research (ANSIR) Laboratory, University of Texas Southwestern Medical Center, Dallas, TX 75390, USA; 2 Department of Radiology, University of Texas Southwestern Medical Center, Dallas, TX 75390, USA; 3 Department of Biomedical Engineering, Wake Forest School of Medicine, Winston-Salem, NC 27157-1088, USA; 4 Virginia Tech – Wake Forest School of Biomedical Engineering, Wake Forest School of Medicine, Winston-Salem, NC 27157-1088, USA; 5 Center of Injury Biomechanics, Wake Forest School of Medicine, Winston-Salem, NC 27157-1088, USA; 6 Department of Radiology–Neuroradiology, Wake Forest School of Medicine, Winston-Salem, NC 27157-1088, USA; 7 USC Stevens Neuroimaging and Informatics Institute, Keck School of Medicine, University of Southern California, Los Angeles, CA 90032, USA; 8 Division of Pediatric Neuropsychology, Children’s National Health System, George Washington University School of Medicine, Rockville, MD 20850, USA; 9 Translational Science Institute, Wake Forest School of Medicine, Winston-Salem, NC 27157-1088, USA; 10 Childress Institute for Pediatric Trauma, Wake Forest School of Medicine, Winston-Salem, NC 27157-1088, USA

**Keywords:** contact sports, diffusion imaging, functional imaging, head impacts, MEG, MRI, neurocognitive testing, repetitive head impacts, sport-related head impacts

## Abstract

The effect of repeated subconcussive head impacts in youth and high school sports on the developing brain is poorly understood. Emerging neuroimaging data correlated with biomechanical exposure metrics are beginning to demonstrate relationships across a variety of modalities. The long-term consequences of these changes are unknown. A review of the currently available literature on the effect of subconcussive head impacts on youth and high school-age male football players provides compelling evidence for more focused studies of these effects in these vulnerable populations.

## Lay abstract

Concussions are known to cause clinical symptoms, which are especially concerning for youth and high school athletes. However, the effects of repeated head impacts that do not cause a diagnosed concussion, known as subconcussive head impacts, are currently unknown. Recent research has identified similar changes in the brain following repeated nonconcussive impacts to the head, once thought to be caused only by the occurrence of concussion with the presence of clinical symptoms. Similarly, many reports suggest that a higher exposure to head impacts is associated with a greater amount of structural and/or functional changes in the brain. Given the similar effects on the brain, with or without symptoms, more work is needed to determine the long-term effects of subconcussive head impacts on individual athletes, particularly in the youth and high school age population.

Concussion is a term used to summarize the set of signs and symptoms following force to the head or body, resulting in brain movement. Many questions remain unanswered regarding the pathophysiology of this injury, the effects on brain function and the long-term effects of multiple concussions. There is also a paucity of literature on impacts to the head that do not result in measurable signs and symptoms that lead to a clinically diagnosed concussion [1,2]. The impacts to the head not resulting in a clinically diagnosed concussion, used heretofore as ‘subconcussive head impacts’, may warrant similar attention in clinical care and research as impacts resulting in a concussion. Subconcussive head impacts are often repetitive and the result of regular athletic play. The number of impacts that a single player sustains over the course of a single season far exceeds the incidence of concussion [3]. These subconcussive head impacts have been historically overlooked because the athlete appears to be unaffected. Early evidence suggests that there may be a link between subconcussive head impacts and functional changes such as memory and cognitive impairments and depression, as well as brain alterations similar to those in neurodegenerative diseases including dementia and Parkinson’s diseases.

Biomechanical head impact data describes the exposure dose hypothesized to cause structural and functional changes in the brain [4,5]. Quantifying the nature and extent of head impact data allows researchers to understand the biomechanical basis of concussive and subconcussive head impacts. Subsequent brain imaging can measure change to structure and function. Many newer imaging techniques can measure more subtle effects such as blood flow changes, brain white matter (WM) integrity and neuronal electrical activity. Functional effects, such as those seen through cognitive assessments, can measure the functional output of the brain.

Neurocognitive tests and neuroimaging are methods of discerning the functional and structural changes in the brain. While clinically diagnosed concussion normally does not show findings on conventional neuroimaging, such as computed tomography or structural MRI, there is an increasing body of literature documenting the microstructural changes in the brain that occur following concussion using advanced imaging techniques. Recent studies have shown that subconcussive head impacts can alter the microstructure of the brain without causing clinically apparent symptoms (i.e., concussion) [1]. To date, only a small number of studies have utilized imaging and neurocognitive testing to investigate the changes in the brain that can occur following a single season of contact sport in the absence of a clinically diagnosed concussion [5–7].

A recent topic of interest behind sports head injury research is the recent case reports of chronic traumatic encephalopathy (CTE) in deceased athletes with a known history of participation in collision sports at the youth, high school, collegiate and/or professional level. CTE is a neurodegenerative disease of varied symptoms including depression, cognitive and/or neurobehavioral dysfunction (for a review of the disease, see Constanza *et al.*) [8]. However, currently CTE can only be diagnosed postmortem via autopsy of the brain, the key finding being a specific pattern of accumulation of tau proteins [9]. CTE is listed as a long-term consequence of repetitive concussion and subconcussive head impacts [10]. There have been recent studies suggesting a greater degree of long-term damage in white mater tracts, as well as a greater degree of cognitive impairment in retired National Football League players who were exposed to tackle football before the age of 12 years[11,12]. However, a subsequent study, using different statistical methods, did not find associations between years of exposure to pre-high school football and neuroradiological, neurological or neuropsychological outcomes [13]. These studies raise significant concern on whether participation in collision sports at a young age causes potential functional and structural changes over the course of a single season and/or multiple seasons and if these changes have long-term negative neurological effects.

This manuscript is a review of the literature documenting existing biomechanical correlates of head impact exposure as well as reported changes in the research setting in functional MRI (fMRI), arterial spin labeling (ASL), diffusion tensor imaging (DTI) of WM, diffusion kurtosis imaging (DKI), magnetoencephalography (MEG) and neurocognitive evaluation that occur over a single season of play in the absence of a clinically diagnosed concussion. In particular, the review focuses on studies of subconcussive head impacts in youth and high school-age male football players, where the youth age group has been traditionally under-represented in collision sports studies.

## Biomechanics

Biomechanical metrics describe an athlete’s exposure to head impacts. The metrics selected for analyses are important for interpreting and understanding pre to postseason changes in neuroimaging and neurocognitive data. When studying the changes due to head impacts, the biomechanical metrics represent the exposure, or dose, measurement when evaluating clinical outcome measures after some period of time. With the advent of head-impact sensing devices, researchers are now able to collect on-field head impact data in real-time and describe head impact exposure of an athlete and/or population. Previous studies characterizing on-field head impact measurements in football have shown that male football athletes are exposed to a wide range of impact severities (10–120+ g’s of linear acceleration and <10–6000+ rad/s^2^ of rotational acceleration). These studies also give an estimate of the number of impacts received per practice, per game, per season and per level of play [14–18]. Much of the biomechanical research has been focused on football and has been performed at the collegiate level. Recent studies at the youth and high school levels of football have demonstrated that the frequency and severity of head impacts increases with each level of play. However, younger athletes participating in tackle football are still exposed to high magnitude head impacts, albeit at a lower frequency than high school or collegiate athletes [14,17–19].

The total number of impacts an athlete receives over the course of a season has been suggested as a simplified exposure metric and has been correlated with neuroimaging [6,20–21], neurocognitive testing [22,23] and balance assessment [22] measures. These correlations are discussed in more detail later in this manuscript in their respective sections. Many studies have found total number of impacts to be significantly correlated with region of interest-based imaging metrics [20], change in balance performance measured on the balance error scoring system total error score [22], change in WM integrity measured with DTI [24] and cognitive impairment [6]. A recent study by Bazarian *et al.* however, did not find relationships between total number of impacts and DTI metrics [21]. The number and severity of impacts experienced in the final weeks (14 days) prior to postseason assessment have been found to have an effect on DTI [20], fMRI [25] and cognitive measures [23]. While counting impacts offers a basic understanding of head impact exposure in terms of quantity, it does not fully describe cumulative head impact exposure as the distribution of the direction and magnitude of head accelerations measured during a season often differs for two athletes with the same total number of impacts.

Many biomechanical studies characterize the head impact exposure of an athlete or a population by the median, 95th percentile and maximum peak linear and rotational resultant acceleration. Previous studies have shown that on-field head impacts measured in football heavily cluster around impacts with a peak linear resultant acceleration magnitude between 18–22 g’s, with fewer impacts at higher magnitudes. The median and 95th percentile head impact measured in football generally increase with each level of play (youth to collegiate) [18], however there is often variability in player-specific impact distributions [14,17]. Studies by McAllister *et al.* and Bazarian *et al.* have found season peak linear resultant acceleration, the highest peak linear resultant acceleration recorded for a single athlete, to be correlated with neurocognitive metrics [23] and changes in WM integrity measured from DTI [26], respectively. Additionally, McAllister *et al.* have found season 95th percentile peak resultant linear and rotational acceleration to be correlated with region of interest-based DTI metrics [20], however no other studies have significant associations of the aforementioned metrics with neurocognitive or neuroimaging measures. Although these metrics, particularly the 95th percentile impact magnitude, are valuable in describing the severity of impacts an athlete is exposed to, they do not account for the quantity of impacts received by an athlete, which is important when considering the total exposure of an athlete.

The location of impact may be one important variable in head impact exposure. Previous studies by Talavage *et al.* and Breedlove *et al.* have evaluated cognitive impairment by impact location and have found a greater number of top-front impacts in those that are functionally impaired compared with those not demonstrating functional impairment [6,25]. These studies however, did not account for the combined effects of impact location and impact magnitude on cognitive function. The head impact telemetry system (HITS; sideline response system, Riddell, OH, USA) is most widely used by researchers to collect on-field head impact data in football [3,15–16,19,27–34]. It consists of a sideline base unit with a laptop computer connected to a radio receiver and an encoder unit for each football helmet. This system collects impact data on the sidelines from each encoder equipped with six single-axis accelerometers. HITsp is a metric computed by the HIT system which incorporates impact location into the exposure measurement. HITsp is computed by combining peak linear and peak rotational resultant acceleration into a single metric using principal component analysis and is weighted based on impact location [35]. This metric is computed on a per-impact basis and is often evaluated using summary statistics describing the severity of HITsp, including median, 95th percentile and maximum value, as well as a summation of HITsp for each athlete. McAllister *et al.* has found season 95th percentile HITsp to be significantly correlated with imaging metrics measured from DTI suggesting the importance of impact magnitude and direction on the response of the brain [20]. Additionally, McAllister *et al.* found peak HITsp on the last day of play as well as peak HITsp and summed HITsp during the final week of play to have an association with neuropsychological measures suggesting the need to consider the head impact exposure experienced prior to postseason evaluation [23].

Recent efforts have been made to identify athletes that have experienced high magnitude head impacts. Researchers have evaluated changes in neuroimaging and neurocognitive data with respect to the number of head impacts received by an athlete above a certain peak linear and/or rotational acceleration threshold [6,21–22]. Various thresholds have been evaluated ranging from 50 to 200 g’s [21] and 1500 and 6000 rad/s^2^ [21], for linear and rotational acceleration, respectively. Bazarian *et al.* reported a trending relationship between the number of head impacts a football player received above 50 g’s, 100 g’s, 150 g’s, 3000 rad/s^2^, 4500 rad/s^2^ and 6000 rad/s^2^ and changes in fractional anisotropy (FA), however due to the small number of athletes (n = 10), it is difficult to know if these findings are reproducible in larger sample sizes [21]. A threshold-based metric is a method to isolate athletes exposed to large numbers of high magnitude head impacts, however there is often no statistical basis for threshold selection, nor does it account for the cumulative exposure of the athlete.

Efforts to develop a cumulative metric that incorporates the entire season of head impacts have been made. These include summed acceleration [16] and risk weighted cumulative exposure (RWE) [14]. The summed acceleration proposed by Broglio *et al.* directly sums the linear or rotational accelerations experienced for each athlete. Higher summed acceleration has been associated with a worse balance performance score [22]. Additionally, McAllister *et al.* showed sum of linear acceleration and sum of rotational acceleration on the last day of play in the football season were each significantly correlated with neurocognitive testing scores, particularly impulse control, reaction time and total symptom score [23]. Although this method captures head impact exposure, accounting for both the severity and frequency of impacts, in aggregate, it does not take into consideration the nonlinear relationship between peak resultant acceleration and risk of concussion defined by Rowson *et al.* using logistic regression [15,36–37].

Recently, Urban *et al.* proposed a biomechanical metric which weights impacts based on the risk of concussion for linear or rotational acceleration separately (RWE_Linear_ and RWE_Rotational_), or for the combined risk of the linear and rotational components of each impact (RWE_CP_) [14]. This metric weighs the severity of each head impact experienced by an athlete over the course of a season by the risk of concussion associated with the peak acceleration values. This results in a higher weight for higher magnitude head impacts. The risks are subsequently summed to result in a single metric. RWE may provide a better quantification of the cumulative head impact exposure of an athlete as it incorporates the number of impacts and nonlinear relationship between impact magnitude and concussion risk. A previous study by Davenport *et al.* found the RWE_CP_ and RWE_Linear_ to be strongly correlated with changes in DTI measures in high school football players [24]. While RWE has promise for capturing the cumulative head impact exposure of an athlete, it and many of the previously mentioned metrics does not account for the directional dependence on brain injury risk, nor the effects of previous exposure on brain injury risk.

Finite element (FE) modeling is a technique in which kinematic data over time are used to simulate an impact or several impacts using a mathematical model with representative geometry and material properties of the brain. This allows a user to evaluate the strain response relative to the duration, direction and magnitude of impact. Several biomechanical metrics to date have been based on summary statistics (peak, median, 95th percentile linear and/or rotational resultant acceleration); however previous studies involving FE modeling of head impacts have demonstrated that the response of the brain is directionally dependent and duration-dependent [38–40]. For example, Weaver *et al.* reported increased cumulative strain damage measure, a measure of the volume of the brain exceeding a user-defined strain threshold, in the cerebrum and brain stem when the brain is rotated about the transverse and sagittal axes, respectively [38,41]. The development of a strain-based metric measured by modeling each head impact in aggregate with an FE head model may result in stronger correlation with clinical outcome measures than kinematic-based measures alone.

There are a number of biomechanical variables that can be related to potential neuroimaging and neurocognitive changes observed in the brain after impact exposure. These include summary statistics of linear and rotational accelerations, number of impacts, time between impacts, impact duration, impact location and prior head impact exposure. To date, there is no standard biomechanical metric of head movement which best describes the exposure of an athlete to head impacts. Although the biomechanical metric selected for analysis is dependent on the research question, it is important to develop and standardize the use of age-appropriate, and sport-specific, metrics which capture the cumulative subconcussive head impact exposure for use in studying the relationship between head impacts and changes in brain function and structure.

## Neuroimaging

Neuroimaging has begun to play a critical role in measuring the effects of subconcussive head impacts. MRI is a noninvasive tool that can provide a broad range of measurements of the potential functional and structural changes in the brain. Two functional methods are discussed: blood oxygen level dependent (BOLD) and ASL. Diffusion imaging, including DTI and DKI, are structural methods included in this review. MEG is a separate form of functional imaging which is also discussed.

### Blood oxygen level dependent fMRI

BOLD fMRI measures fluctuations of cerebral blood flow (CBF) oxygenation level in the brain associated with neural activity [42]. Even in the resting state, the brain is highly active; such that resting state fMRI has revealed a number of consistent brain networks in healthy subjects. There has been a growing interest in resting state network analyses to explore neuronal changes associated with neurological and psychiatric diseases [43]. Due to its relation with neural activity, BOLD fMRI has recently been used to identify subtle alterations in brain function created by subconcussive head impacts in high school football players [7,44–47].

Abbas *et al.* investigated whether cumulative subconcussive head impacts in high school football athletes without a history of clinically diagnosed concussion was associated with differences in resting state fMRI connectivity of the default mode network (DMN). The DMN is a network of brain regions where the activity is highly correlated in wakeful rest, or when day-dreaming. DMN interconnectivity analysis was performed by computing the correlation between fMRI time series of a seed, or point, placed in the posterior cingulate/precuneus (a main node in the DMN) and that of automated anatomical labeling atlas regions [48]. A hyperconnectivity pattern of DMN was demonstrated for football athletes compared with noncollision-sport controls in most scanning sessions, including followup and preseason which may indicate brain function alterations placed in the posterior cingulate/precuneus (a main node in the DMN) and that of automated anatomical labeling atlas regions [48]. The difference between football athletes and controls at preseason may indicate brain network alterations have accumulated over years of playing the collision-sport [46]. The hyperconnectivity of the DMN has been described as a brain plasticity effect to compensate for neuronal injury, related to the subconcussive cumulative head impacts [46,47]. However, this has only been shown as a correlation, and no studies have shown subconcussive impacts to directly cause hyperconnectivity of the DMN [46,47].

In a working memory fMRI study on a cohort of high school football athletes with no clinically observed symptoms of concussion, a significant relationship was reported between the fMRI activity changes (from preseason to in-season followup scans) and the number of head impacts as measured by the HIT system [7]. The spatial distribution of the activity changes was mainly in the frontal and cerebellar regions as well as occipital visual areas.

Significant alterations of working memory fMRI activity during in-season assessments have been found in dorsolateral prefrontal cortex for nonconcussed high school football athletes who exhibited abnormal performance on the cognitive tests when compared with those who had normal performance [44]. This finding was augmented by the observation that these athletes sustained higher numbers of head impacts on the top-front of their head. These findings may suggest that subconcussive head impacts may result in neuronal injury and subsequent cognitive impairment, measured by fMRI, which cannot be identified clinically [44].

### ASL

ASL is a completely noninvasive form of perfusion MRI used primarily to directly measure CBF [49]. It is often also classified as fMRI, due to the relationship of perfusion with brain function. It differs from BOLD imaging, in that ASL is a direct measure of CBF, and BOLD is a product of the extraction fraction of oxygen from local capillaries and blood flow. ASL does not require a contrast agent in order to measure blood flow. Water in the blood is labeled magnetically and then imaged. While studies have yet to show ASL measurable changes related to subconcussive head impacts, some studies have shown changes related to clinically diagnosed mTBI. Maugans *et al.* found decreases in CBF following concussion in youth athletes [50]. Conversely, Militana *et al.* found increased CBF after diagnosed concussion in a group of collegiate athletes compared with controls [51]. ASL is a fairly new imaging method and area of research, which requires more studies to understand the relationship between subconcussive head impacts on blood flow, particularly in younger athletes.

### MEG

MEG is a noninvasive form of brain imaging that detects magnetic fields induced by neuronal electrical activity, with millisecond time scale resolution [52,53]. MEG is ideal for measuring functional changes in the brain due to its high temporal resolution. MEG also measures functional changes in the brain in an inherently different way than fMRI or ASL, which may allow it to be more sensitive to certain diseases or injuries with functional changes [54]. MEG has been used to identify changes in the brain following concussion [55]. These changes are often seen as increases in the delta, or low-frequency, band after injury. Delta waves are low-frequency brain signals ranging from 1 to 4 hertz. These low-frequency waves are considered pathologic in normal, alert adults and have been previously associated with concussion [55]. Huang *et al.* measured increased delta waves in 87% of the 45 adult patients with concussion from combat related blasts as well as nonblast causes [55]. However, no studies to date have been published relating MEG data to subconcussive head impacts.

### DTI

DTI is an MRI technique. DTI can detect the Brownian motion of water molecules through the use of motion sensitizing gradients [56]. DTI uses at least six gradient directions, which allows diffusion to be modeled anisotropically, or dependent on direction. DTI gives the ability to perform fiber tracking and derive a diffusion tensor and scalar metrics. Standard scalars are FA and mean diffusivity (MD). FA describes the degree of anisotropy of a diffusion process. An FA value of zero represents equal movement of molecules in all directions (i.e., isotropic diffusion); whereas an FA value of one represents movement entirely in one direction [57,58]. FA can be separated into linear (C_L_), planar (C_p_) and spherical (C_s_) anisotropy components to further explain the shape of the diffusion ellipsoid [59,60]. These diffusivity metrics allow a closer examination of the microstructural causes of abnormal FA. Linear anisotropy represents diffusion along two orthogonal directions, also referred to as ‘cigar’ shaped. Major WM tracts, like the corpus callosum, have high linear diffusivity. Planar diffusion is restricted to a plane (or saucer-shaped). Planar diffusivity is often associated with crossing, or ‘kissing’, fibers. Spherical diffusion is purely isotropic diffusion. The highest spherical measures are seen in CSF, where diffusion is unbounded [61].

Previous studies on subjects with concussion have documented changes in DTI measures in regions of the brain such as the corona radiata, corpus callosum, cingulate fasciculus, inferior longitudinal fasciculus, inferior fronto-occipital fasciculus, internal capsule, external capsule, corticospinal tracts, fornix and the hippocampus. This has been discussed in detail in previous DTI focused reviews [62–65]. Studies on the effects of subconcussive head impacts, including those by Bazarian and Davenport, have revealed a similar distribution of WM abnormalities as previously discussed [5,21,66–67].

The direction of change in FA and MD following subconcussive head impacts may be dependent on the time point at which the subjects are imaged [66,68]. DTI of the whole brain performed at the end of a season of play represents a snapshot of all of the brain regions that sustained injury over the course of the season. Areas of the brain that were injured at the beginning of the season may be in a different stage of healing than those injured near the end of the season. Bazarian *et al.* found subject-specific concomitant increases and decreases in FA and MD in different brain regions, which they speculated was due to multiple axonal injuries separated spatially in the brain and temporally across the 3-month football season [26]. While not in a youth population, these findings support the dynamic nature of axonal injury, even over a single season of football without a clinically diagnosed concussion.

Additional DTI metrics include axial diffusivity (AD) and radial diffusivity (RD). AD describes the magnitude of diffusion that occurs parallel to the primary orientation of the diffusion process. In WM, AD measures diffusion parallel to the axon and is thought to be sensitive to changes in axonal integrity [69]. RD describes the magnitude of diffusion that occurs perpendicular to the primary orientation of the diffusion process. In WM, RD measures diffusion perpendicular to the axon and is thought to be sensitive to changes in myelination [69]. However, changes in AD and RD have not yet been evaluated with respect to head impact exposure in youth or high school football and should be explored in the context of subconcussive head impacts.

### DKI

DTI uses a linear estimation method, which is only able to estimate the Gaussian diffusion. DKI is a novel extension of diffusion imaging. DKI can calculate the diffusion in a nonlinear fashion to capture the non-Gaussian components of diffusion in the brain. Kurtosis is a dimensionless measure of the deviation from a Gaussian, or normal distribution. A greater degree of structure in the tissue through which water is diffusing causes more deviations from Gaussian movement, and, therefore, increases the excess diffusional kurtosis measurement [70]. DKI-derived metrics primarily include mean kurtosis (MK), axial kurtosis (K axial) and radial kurtosis (K radial). Due to the ability of DKI to accurately model the non-Gaussian water diffusion in the brain, the DKI measures and modeling approaches may provide additional information on the pathophysiologic mechanisms underlying WM injury related to subconcussive head impacts.

Stokum *et al.* and Grossman *et al.* showed decreases in MK in subjects with concussion when compared with controls [71,72]. Another study demonstrated changes in several DKI-derived scalars that correlated with head impact exposure after a season of high school football [73]. In an animal study, Zhuo *et al.* showed increases in MK, from baseline, which coincided with increases in K axial and decreases in K radial [74]. DKI may provide different information regarding WM integrity than previously attainable with DTI.

### Imaging summary

Advanced imaging analysis shows promise for identifying changes in the brains of athletes who have experienced repetitive subconcussive head impacts. DTI and fMRI have the most supporting literature to date in youth and high school-age population studies. ASL, MEG and DKI may also provide useful information (Table 1).

**Table 1.** **Summary of imaging findings after one season of subconcussive impacts in contact sports.**

**Modality**	**Metric**	**Type of change**	**Literature**	**Ref.**
fMRI	BOLD	Altered network	Abbas *et al.* (DMN); Talavage *et al.* (working memory)	[46,47]
	ASL	Expected decreases or increases	None known yet for subconcussive; Maugan *et al.* (decrease TBI); Militana *et al.* (increase TBI)	[51]

MEG	Delta waves	Increases	None known yet for subconcussive; Huang *et al.* (concussive)	[55]

DTI	FA	Increases and/or decreases	Bazarian *et al.*; Davenport *et al.*	[5,21]
	MD	Increases and/or decreases	Bazarian *et al.*	[21]

DKI	MK and others	Increases and/or decreases	Davenport *et al.*	[73]

ASL: Arterial spin labeling; BOLD: Blood oxygen level dependent; DKI: Diffusion kurtosis imaging; DMN: Default mode network; DTI: Diffusion tensor imaging; FA: Fractional anisotropy; fMRI: functional magnetic resonance imaging; MEG: Magnetoencephalography ; MK: Mean kurtosis; TBI: Traumatic brain injury.

## Neurocognitive testing

Cognitive testing is an integral part of assessing brain functioning after concussion. The strongest effect sizes and changes in performance are often seen acutely after injury. Concussion diagnosis is aided by patient reported symptoms and cognitive function deficits using tools such as the SCAT3 [75]. Conventional clinical imaging, such as computed tomography or structural MRI, often show no visible results. The cognitive tests that are most sensitive in postconcussion assessment measure the individual’s short term and working memory, information processing speed, attention, concentration and reaction time [76,77]. These tests may either be administered at the sideline or following a concussive or subconcussive impact. They measure immediate change in brain functioning, along with orientation and symptom status.

One of the more widely used tests for ongoing cognitive and symptom effects after concussion is the Immediate Post-Concussion Assessment and Cognitive Testing (ImPACT; ImPACT™, PA, USA), which was designed specifically to assess concussion symptoms and cognitive deficits in athletes [78,79]. Breedlove *et al.* measured head impacts of high school football players using the HIT system and evaluated cognitive function preseason, in-season and postseason using the ImPACT (ImPACT™) [25]. They found that athletes without clinically observed symptoms, but with a functionally observed impairment (defined as changes in fMRI or neuropsychological testing) had more head impacts than those athletes without either clinically or functionally observed impairment. They also studied the effect of head impact location and found that players without a clinically observed symptoms, but with a functionally observed impairment had significantly more side head impacts than those players with a clinically observed impairment. Talavage *et al.* performed a similar study including 11 high school football players with HITs and ImPACT data [6]. The players with no clinically observed findings, but with functionally observed findings on imaging, had significantly greater head impacts to the top front of the head. Davenport *et al.* also studied a high school football team over a single season using the HIT system and administering the ImPACT pre- and post-season. They found that decreases in Verbal Memory composite score from pre- to postseason correlated with the cumulative head impact exposure metric, RWE [5].

## Conclusion & future perspective

Biomechanical metrics provide researchers the ability to measure the exposure of an athlete to head impacts. There are various biomechanical metrics which have been developed, however there is a need to develop improved metrics and standardize their use in order to better understand the biomechanical basis of the effects of subconcussive head impacts. In addition, there are numerous imaging modalities available to researchers interested in the effects of impacts to the head, including those that do not result in a clinically diagnosed concussion. Currently, the modalities with the most evidence in the literature for showing changes related to subconcussive head impacts include DTI and fMRI. Additional modalities, such as MEG and DKI, may be more sensitive and specific to subconcussive head impacts and should be researched further. Future work should consider the sensitivity of neurocognitive tests to minor changes in behavior or cognitive ability. There is a notable relative lack of research in these areas with youth and to a lesser degree high school-age populations. In addition, much of the current research focuses only on male football players. It is vital to study populations of all ages and genders across all sports in order to better understand the effects of head impacts across development. A critical aspect in establishing these relationships is the availability of robust biomechanical metrics. Another significant consideration for subconcussive research is the underreporting rate for concussions. Correct and timely identification of a concussion can be improved by education of players, coaching staff and parents as well as the presence of an athletic trainer and in-season neuropsychological testing. It is important to note that the functional outcomes, duration and consequences of the imaging changes related to subconcussive head impacts are not well understood. Careful large-scale longitudinal studies will be necessary to determine the longer term effects of many of these imaging-based alterations.

**Executive summary**

**Background**

- There is increasing evidence showing the potential link between repetitive head impacts and alterations in brain structure and function.
- Functional MRI, diffusion tensor imaging and neurocognitive testing are tools which have been used to evaluate changes that occur in a single season in relation to biomechanical correlates.

**Biomechanics**

- On-field collection of kinematic head impact data allows researchers to quantify head impact exposure based on various metrics. Biomechanical metrics have been used to study pre to postseason changes in imaging and cognitive measures.
- Various metrics have been found to correlate with imaging and cognitive metrics measured over a single season, however there is no standard biomechanical metric which best describes the head impact exposure of an athlete to subconcussive and concussive head impacts.

**Imaging**

- Conventional neuroimaging is typically unremarkable in cases of clinical concussion and long-term subconcussive damage.
- Advanced diffusion and functional imaging metrics have been shown to statistically correlate with the number and severity of subconcussive head impacts at the group level. Whether these metrics increase or decrease is debated and often dependent on time of injury in relation to imaging.

**Neurocognitive testing**

- Neurocognitive testing and structured symptom assessment aids in the diagnosis of concussion for individual patients in an acute setting and has been used to measure changes in cognitive functioning following injury.
- A variety neurocognitive outcomes have been found to statistically correlate with the number and severity of subconcussive head impacts at the group level. However, the literature is mixed.

**Conclusion & future perspective**

- There are numerous imaging modalities available to researchers interested in the effects of subconcussive head impacts.
- The availability of robust biomechanical data and biomechanical metrics is critically important for understanding the changes associated with subconcussive and concussive head impacts.
- The functional outcomes, duration and consequences of the imaging changes related to subconcussive head impacts are not well understood.
- The majority of studies have been focused on collegiate populations and little is known about how these changes translate to youth and adolescent athletes.
- Careful large-scale longitudinal studies, using multiple imaging modalities, will be necessary to determine the longer term effects of subconcussive impacts on the structure and function of the brain.
